# Predicted impact of lipid lowering therapy on cardiovascular and economic outcomes of Swedish atherosclerotic cardiovascular disease guideline

**DOI:** 10.1186/s12872-017-0659-2

**Published:** 2017-08-16

**Authors:** Gunilla Journath, Kristina Hambraeus, Emil Hagström, Billie Pettersson, Mickael Löthgren

**Affiliations:** 1 Cardiology unit, Department of Medicine, Karolinska Institutet, Karolinska University Hospital, Stockholm, Sweden; 2 Department of Cardiology, Falun Hospital, Falun, Sweden; 3 Uppsala Clinical Research Center, and Department of Medical Sciences, Uppsala University, Uppsala, Sweden; 4 Amgen Inc, Thousand Oaks, California, USA; 5 Amgen (Europe) GmbH, Zug, Switzerland

**Keywords:** Cardiovascular disease, Costs, Guidelines, Lipids, Myocardial infarction

## Abstract

**Background:**

The effects on cardiovascular disease (CVD) by treatment recommendations on prevention of atherosclerotic CVD remain to be evaluated. The objectives were to assess treatment gap for low density lipoprotein cholesterol (LDL-C) according to guidelines, potential impact on CVD outcomes, and possible avoided economic costs, in post myocardial infarction (MI) patients, if target LDL-C levels of ≤1.8 mmol/L would be achieved.

**Methods:**

All patients registered in the Swedish Secondary Prevention after Heart Intensive care Admission register, with one-year post-MI follow-up during 2013 were selected. The REACH risk prediction and a calibrated model for recurrent cardiovascular events and death were used to estimate unadjusted risk prediction based on the REACH equation henceforth called base case, and calibrated CVD outcomes based on gender-specific risk factors. The predicted impact of the LDL-C reduction on the risk of CVD was based on the Cholesterol Treatment Trialists´ Collaboration findings.

**Results:**

A sample of *n* = 5904 patients (74% men) with a mean age of 64 years were included. Around 70% did not reach LDL-C target ≤1.8 mmol/L. Over a 10-year period, 820–2262 events were predicted to occur in those who did not reach target corresponding to 20% – 55% risk of CVD events. To achieve LDL-C target, the mean LDL-C had to be reduced by 0.73 mmol/L (29%). If this LDL-C reduction was achieved, 195–544 life years, 132–343 CVD events, and 7.9–20.9 million Swedish crowns (MSEK) of direct costs, and 19.3^−^51.0 MSEK of total costs would be avoided.

**Conclusion:**

Lowering of LDL cholesterol to achieve target levels according to guidelines for post-MI patients may lead to fewer cardiovascular events and avoidance of event costs.

## Background

Ischemic heart disease (IHD) is the most common CVD, and the leading cause of death in large parts of the world [1] Reduction of low-density lipoprotein cholesterol (LDL-C) with lipid lowering therapy (LLT) has shown a reduction of the risk of cardiovascular events in both high and low risk individuals [2–4]. Meta-analysis of statin trials observed that further risk reductions were found in patients obtaining LDL-C levels below 1.8 mmol/L [5, 6].

European guidelines recommend treatment target levels of LDL-C depending on predicted risk for cardiovascular events, with lower target levels for patients at high risk (very high risk: <1.8 mmol /L; high risk <2.5 mmol/L; moderate risk <3 mmol /L) [7]. Guidelines from the US have another approach recommending fixed-dose strategies instead of targeted goals to lower blood cholesterol [8]. High intensity statin therapy was recommended for patients with high or very high risk, and a low dose statin therapy to those with moderate risk of cardiovascular disease [8].

The Medical Product Agency in Sweden published treatment recommendations on prevention of atherosclerotic cardiovascular disease in 2014 [9], with a similar approach as European guidelines with recommended treatment target of LDL ≤1.8 mmol/L for high risk patients. Patients with established coronary artery disease were classified as high-risk patients in all CVD prevention guidelines. The risk of recurrent disease remained high despite modern treatment for myocardial infarction [10]. Treatment with lipid-lowering agents is cost-effective, especially in high-risk patients [11, 12]. To our knowledge the potential consequences the Swedish guideline have not been published.

The aims were to assess treatment gap for LDL-C according to guidelines, potential impact on CVD outcomes, and possible avoided economic costs, in a cohort of Swedish post myocardial infarction (MI) patients, if target LDL-C levels of ≤1.8 mmol/L would be achieved.

## Methods

### Setting, study design, and study population

SWEDEHEART is a Swedish national register in which patients with acute coronary syndrome are prospectively registered. Patient characteristic, hospital treatments, drug treatments at discharge, and outcome for patients consecutively included and treated at all Swedish coronary care units are collected in this register [13]. The Swedish Secondary Prevention after Heart intensive care Admission (SEPHIA) is a sub register within SWEDEHEART collecting data on secondary prevention and cardiac rehabilitation [14]. Follow-up data were registered by office visits or phone call, supplemented by blood samples collected at the patient’s primary care centre, at six to ten weeks and at 12 to 14 months post MI. Around 80% of all Swedish MI patients below the age of 75 years are included in this register [14].

In this study, a cohort of 5904 patients (74% men) registered in the SEPHIA register and who had one year follow-up during 2013, were included. Data from the SWEDEHEART/SEPHIA national register was extracted in an aggregated form. In accordance with Swedish regulations, written informed consent is not necessary for national registers, however all patients were informed about their participation in the register, and their right to decline participation.

### Prediction of cardiovascular disease risk

The Reduction of Atherothrombosis for Continued Health (REACH) risk function was used to predict 20-month risk of recurrent CVD [15]. This model provided estimates of recurrent non-fatal and fatal CVD events based on the following risk factors: age (years), gender (male/female), smoking, diabetes mellitus; body mass index (BMI) <20 kg/m^2^, number of vascular beds with CVD-manifestations (1, 2, 3), congestive heart failure, atrial fibrillation, statin treatment, and acetylsalicylic acid treatment [15]. The predicted 20-month fatal and non-fatal CVD risk were derived from the “next event” REACH equation using the detailed Cox regression model covariate coefficient estimates provided in Wilson et al. 2012 Appendix [15]. CVD rates were predicted separately for males and females, before calculating a weighted average the overall cohort risk, accounting for gender variation in risk factors. CVD risk was estimated separately for each year in the prediction time period, accounting for the yearly increase in cohort age and the impact of increased age on CVD risk. The predicted 20-months risks were annualized for each year of prediction. The effect on CVD risk derived from the lowering of LDL-C was calculated based on the Cholesterol Treatment Trialists’ Collaboration (CTTC) meta-analyses results, linking LDL-C lowering to CVD event risk reduction [6]. Different rate ratios of CVD event reductions per mmol/L LDL-C reduction were used based on CTTC: MI (0.71), ischemic stroke (IS) (0.69) and fatal CHD (0.80) [6]. For fatal stroke a rate ratio of one was used based on the non-significant difference reported by CTTC [6]. The proportion (%) of non-fatal (MI and stroke) vs. fatal CVD event post MI, was based on Jernberg et al. reported event distribution of up to 24 months post MI: 46.8% CVD death, 37.8% MI, 15.4% stroke [16]. In the age ranges 55–64 to 65–74 years of fatal CHD vs. fatal stroke occurred in 96% vs. 4% post-MI, indicating that fatal CHD is more common than fatal strokes in post MI patients [17]. The direct costs of non-fatal MI and stroke were based on Hallberg et al. 2015 [18] and were for MI: Swedish crowns (SEK) 76,657, and ischemic stroke: SEK 88,790. These event cost estimates were from Table 4 in Hallberg et al. 2016, and from the incremental cost year (day 0–365 days after new CVD event) for the CVD history cohort. The reported cost estimates were converted to SEK using the same exchange rate of 1 € = 8.71 SEK as reported by Hallberg et al. 2015, p. 3. Fatal CVD costs were estimated, and based on Ara et al. 2009 [19] and were for CHD death costs SEK 11,345 (14.8% of MI costs) and stroke death SEK 40,577 (45.7% of stroke costs). Total directs costs of CV events were estimated in a first analysis step. In a second step the total cost including costs of informal care by family and relatives, indirect costs of productivity loss due to premature death, and reduced work capacity ere estimated based on previous findings have shown that direct costs accounted for around 41% of total costs [20].

The REACH risk prediction model was based on participants from around the world with different prior CVD events, not only MI [19]. Results from the UK Clinical Practice Research Datalink (CPRD) calibration analyses indicated that the REACH risk prediction significantly underestimated the risk of CVD events in a post-acute coronary syndrome population [21]. Analyses of the REACH risk prediction were therefore, calibrated according to CPRD analyses. The CPRD study included heart failure (HF) in addition to MI, stroke and CVD mortality outcomes, and hence the reported calibration factor of 3.36 for a post- acute coronary syndrome (ACS) population had to be adjusted for the purpose of this study. Based on the post-ACS cohort, the adjusted calibration factor was 3.06 = 3.36*(1–0.089) in the CPRD cohort accounting for HF incidence in patients between 64 and 73 years old. In addition to the prediction of fatal and non-fatal CVD event using the REACH risk prediction, the predictions account for Swedish age- and gender specific non-CVD mortality life tables from Statistics Sweden were used (available at www.scb.se/hitta-statistik).

### Statistical analyses

Demographics and other baseline characteristics were presented for the overall study population, as well as for the controlled cohort (LDL-C ≤ 1.8 mmol/L), the non-controlled (LDL-C > 1.8 mmol/L) cohort, and in men and women separately. The REACH risk function was used to predict the CVD risk [15], as well as the calibrated CVD risk prediction described above [19]. The possible avoided costs were based on cases at baseline in REACH (henceforth called base case), the calibrated (scenario) risk predictions, the corresponding population’s CVD costs and potential cost reductions linked to LDL-C reduction according to guidelines. This was predicted by combining event prediction and estimation of health care costs associated with each type of event.

## Results

**Fig. 1:**
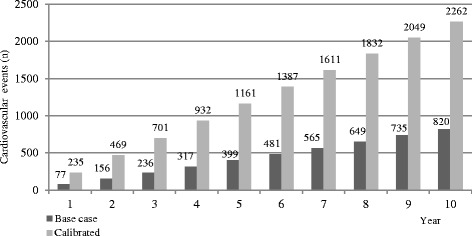
Predicted number of cardiovascular events in base-, and calibrated cases over a ten year period

**Fig. 2:**
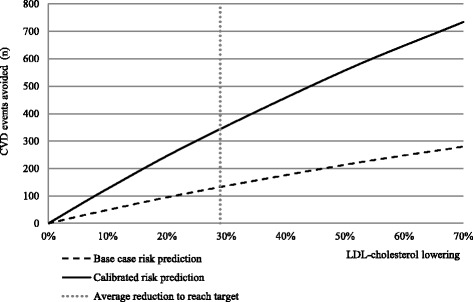
Predicted number of cardiovascular events avoided by percent LDL-C reduction in base-, and calibrated cases over a ten year period

**Fig. 3:**
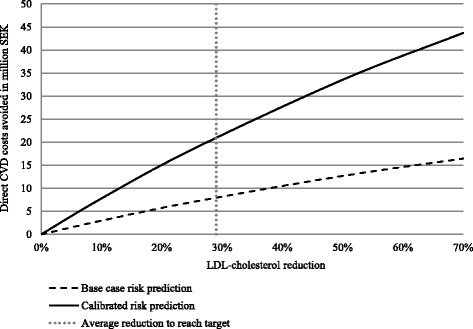
Predicted direct cardiovascular events costs avoided by percent LDL-C reduction in base-, and calibrated cases

Table 1 shows the characteristics of the study population (*n* = 5904, 74% men). Around 70% of the overall cohort did not reach the target of LDL-C ≤ 1.8 mmol/L (men 69% and women 75%). An average LDL-C reduction of 0.73 mmol/L (men 0.70 mmol/L, women 0.81 mmol/L) was required to achieve the LDL-C target corresponding to an average LDL-C reduction of 29% (men 28% and women 31%). There was a lower proportion of patients with diabetes, and statin-treated patients, in the non-controlled group than in the controlled group (Table 1). The base case and calibrated risk predictions ranged from 820 to 2262 total CVD events over a 10-year period in the non-controlled group, corresponding to a baseline CVD event risk of 20% -55% (Fig. 1). Over a ten-year period, the LDL-C reductions to reach target was predicted to lead to 195–544 gained life years, 132–343 fewer CVD events (fatal [39–97], non-fatal MI, and stroke [93–246]) (Fig. 2). The corresponding total direct health care costs were predicted to be reduced by 7.9–20.9 (million Swedish crowns) MSEK and total health care costs by 19.3–51.0 MSEK (Fig. 3).

**Table 1:** Characteristics of the study population

Variable	Overall cohort			Controlled cohort (LDL-C ≤ 1.8 mmol/L)			Non-controlled Cohort (LDL-C > 1.8 mmol/L)		
	Total	Men	Women	Total	Men	Women	Total	Men	Women
n (%)	5904 (100)	4386 (74)	1518 (26)	1759 (100)	1377 (78)	382 (22)	4145 (100)	3009 (73)	1136 (27)
LDL-C, mean (SD) mmol/L	2.2 (0.9)	2.2 (0.8)	2.3 (0.9)	1.4 (0.3)	1.4 (0.3)	1.4 (0.3)	2.5 (0.8)	2.5 (0.8)	2.6 (0.8)
Risk factors for REACH risk predictions									
Age, mean (SD)	64 (9)	63 (8)	65 (9)	64 (9)	64 (9)	64 (9)	64 (8)	63 (8)	65 (8)
Smoking´(%)	13	12	15	13	12	15	13	12	15
Diabetes mellitus (%)	25	24	27	31	30	34	23	22	25
BMI < 20 kg/m^2^ (%)	2	1	4	2	1	4	2	1	3
Number of vascular beds affected	1.1	1.1	1.1	1.1	1.0	1.1	1.1	1.1	1.1
One vascular bed affected (%)	95	95	95	95	96	94	95	95	95
Two vascular beds affected (%)	5	5	5	5	4	6	5	5	5
Congestive heart failure (%)	2	2	2	3	3	3	2	2	2
Atrial fibrillation (%)	3	3	3	4	4	4	3	3	2
Statin treatment (%)	92	93	88	99	99	99	89	91	85
Acetylsalicylic acid treatment (%)	92	93	90	93	93	94	92	93	89

## Discussion

This study shows that around 70% of very high risk patients with prior MI did not have controlled LDL-C, 12 months post MI. We found that an average additional LDL-C reduction by 29% (0.73 mmol/L) would be needed to achieve target level of LDL-C 1.8 mmol/L. This LDL-C reduction was estimated to lead to a total of 132–343 fewer CVD events with corresponding health care costs reduced by in total 19.3–51.0 MSEK, accounting for 20.1% of the total predicted event costs. Over a ten-year period 805–2262 CVD events was predicted to occur in the non-controlled patients (*n* = 4145), corresponding to a 20–55% ten-year risk of non-fatal MI, stroke and fatal CVD event. A total of 195–544 lives were predicted to be gained over ten years in the study population, if target LDL-levels were achieved.

In the western countries the incidence of MI has declined, and one-year post-MI survival has improved [22, 23]. However, patients who have survived a MI, are still at high risk and one in five was estimated to have a recurrent CVD event during a subsequent 10-year year period [16]. A large proportion of patients in this study received LLT but did not reach LDL-C target according to guidelines [9]. Treatment gaps between guidelines and real world results regarding risk factor control for CHD patients in Europe was reported in the EUROASPIRE-studies. Lipid control, defined as LDL <1.8 mmol/L increased from 6.1% in EUROASPIRE II (1999–2000) to 25.6% in EUROASPIRE IV (2012–2013), revealing a failure of current secondary prevention strategies to deliver best possible treatment to patients after a coronary event [24]. Data from the Swedish quality registry SWEDEHEART showed that goal attainment for LDL-C improved, from 46% in 2014 to 51% in 2015 [25]. One explanation for the higher proportion of patients achieving LDL-C goal could be that access to high intensity statins increased, when prices for atorvastatin decreased following the patent expiration. Earlier changes in reimbursement schemes showed that around one fifth of the patients switched from low dose to higher doses of atorvastatin following a new reimbursement scheme, where higher doses of atorvastatin was covered while lower doses were not reimbursed in the new scheme [26]. Non-adherence to prescription may also be a reason for not achieving LDL-C target. Prior observational studies, in patients with IHD, have shown adherence to statin treatment in between 50% to 79% [27, 28]. Factors that may affect adherence could be demographic and socioeconomic factors, side effects, life-style, time since last provider visit and number of pills prescribed [29]. Strategies to improve adherence to secondary prevention medication need to be tailored to relevant patient- and community factors.

The results presented predicted CVD events and costs over a ten-year period for the analysed incident cohort. In order to assess the cost in a given year or over a period of years we needed to assess the CVD events and cost prediction for a prevalent population. This was done based on the available incident cohort and a life time prediction of CVD events and costs underestimated steady-state assumption (ie every year a cohort of same size and characteristics as the study cohorts were assumed to enter post MI). The cohort life-time predictions represented exactly the CVD events and costs expected to materialize in any given year. Under a life-time prediction horizon the base case and calibrated predictions indicated that 1396–2741 life years could be gained, 350–606 CVD events avoided and direct CVD costs reduced 21.4–39.2 MSEK if LDL-C levels were reduced on average 0.78 mmol/L. If willingness to pay for a life year gained is about 553,000 SEK [30, 31] then this would mean that the monetary value of the life years gained in our study would be 108–326 MSEK for the estimated 195–544 life-year gained in base case and risk calibrated scenario [30]. In a sensitivity analysis, using an LDL-C lowering up to 70%, up to 280 CVD events could potentially be avoided (vs case 132 events). Up to 19 M SEK due to CVD costs were estimated to be avoided over a 10-year period in the cohort. Ongoing outcomes studies with other non-statin therapies as PCSK9-inhibitors will add additional information on whether further lowering of LDL-C will prevent CVD events.

### **Limitations and strengths**

The presented analyses and results focused on the assessment of the impact on CVD risk and costs if the current LDL-C target was reached in comparison with the current treatment patterns and practices. CVD risk and costs were predicted over a 10-year time horizon accounting for increased age and consequent increased CVD risk over time. However, the analysis was not accounting for any other risk factor changes over time, and is furthermore not accounting for possible future change of statin treatment goal and attainment rate which may change further with physicians’ care or disease progression. The post-MI population in this study was younger, due to the age limit of the SEPHIA-register, more often revascularised, and had a higher proportion of men, also due to age inclusion criteria, compared with the MI population in general, which limited the generalizability. Since ICD-10 codes were used for morbidity data there was a possibility for coding errors. However, a validation of the Swedish in patient register showed that coding was concurrent in >98% of the cases [32]. The study was based on population averages, and not individual patient-level data. For the analysis of implementation of the guidelines focused on LDL-C this poses some challenges as the guidelines specifies that target LDL-C is ≤1.8 mmol/L, or if this cannot be achieved, a 50% reduction in LDL-C levels. Recently European Guidelines on CVD in clinical practice recommended an LDL-C goal <1.8 mmol/L, or a reduction of at least 50% if the baseline is between 1.8 and 3.5 mmol/L in very high risk patients [33]. The patients in this study represent around 80% of the total MI-population in Sweden in this age group and, potential selection bias was considered to be low. There is a risk of conservative bias, as patients with lower social, financial and health-related functioning may be less likely to attend follow-up visits. The study population should however provide a good estimate of treatment strategies and treatment goals attainment in usual care. Possible future research should compare these risk model based on predictions with actual real-world outcomes over a longer period in the SEPHIA register.

## **Conclusion**

Despite the multitude of evidence of lowering the CVD risk by intensive secondary prevention there was a large treatment gap between guidelines and achievement of target LDL-C. Lowering of LDL cholesterol to achieve target levels according to guidelines for post-MI patients may lead to fewer cardiovascular events and avoidance of event costs.

## Abbreviations

ACS: Acute coronary syndrome
CHD: Coronary heart disease
CPRD: The UK Clinical Practice Research Datalink
CTTC: The Cholesterol Treatment Trialists´ Collaboration
CVD: Cardiovascular disease
EUROASPIRE: European Society of Cardiology survey of secondary prevention of coronary heart disease
HF: Heart failure
IHD: Ichemic heart disease
LDL-C: Low density lipoprotein cholesterol
LLT: Lipid lowering theraphy
MI: Myocardial infarction
MSEK: Million Swedish crowns
REACH: The Reduction of Atherotrombosis for Continued Health
SEK: Swedish crowns
SEPHIA: The Swedish Secondary Prevention after Heart intensive care Admission Swedeheart: Swedish quality registers of coronary heart diseases
US: United States

## References

[1] Mathers CD, Loncar D (2006). Projections of global mortality, and burden of disease from 2002 to 2030. PLoS Med.

[2] Bjorck L, Rosengren A, Bennett K, Lappas G, Capewell S (2009). Modelling the decreasing coronary heart disease mortality in Sweden between 1986 and 2002. Eur Heart J.

[3] Mihaylova B, Emberson J, Cholesterol Treatment Trialists CTT (2012). The effects of lowering LDL cholesterol with statin therapy in people at low risk of vascular disease: meta-analysis of individual data from 27 randomised trials. Lancet.

[4] Zoungas S, Curtis AJ, McNeil JJ, Tonkin AM (2014). Treatment of dyslipidemia and cardiovascular outcomes: the journey so far--is this the end for statins?. Clin Pharmacol Ther.

[5] Boekholdt SM, Arsenault BJ, Hovingh GK (2013). Levels and changes of HDL cholesterol and apolipoprotein A-I in relation to risk of cardiovascular events among statin-treated patients: a meta-analysis. Circulation.

[6] Baigent C, Blackwell L, Cholesterol Treatment Trialists CTT (2010). Efficacy and safety of more intensive lowering of LDL cholesterol: a meta-analysis of data from 170,000 participants in 26 randomised trials. Lancet.

[7] Perk J, De Backer G, Gohlke H (2012). European guidelines on cardiovascular disease prevention in clinical practice (version 2012): the fifth joint task force of the European Society of Cardiology and Other Societies on cardiovascular disease prevention in clinical practice (constituted by representatives of nine societies and by invited experts). Eur Heart J.

[8] Stone NJ, Robinson JG, Lichtenstein AH (2014). 2013 ACC/AHA guideline on the treatment of blood cholesterol to reduce atherosclerotic cardiovascular risk in adults: a report of the American College of Cardiology/American Heart Association task force on practice guidelines. Circulation.

[9] Medical product Agency. To prevent atherosclerotic cardiovascular disease with drugs- treatment recommendation. https://lakemedelsverket.se/upload/om-lakemedelsverket/publikationer/information-franlakemedelsverket/2014/Information_fran_lakemedelsverket_nr_5_2014_webb.pdf. Accessed 16 Oct 2016.

[10] Fox KA, Carruthers KF, Dunbar DR (2010). Underestimated and under-recognized: the late consequences of acute coronary syndrome (GRACE UK-Belgian study). Eur Heart J.

[11] Lindgren P, Graff J, Olsson AG, Pedersen TJ, Jonsson B, Investigators IT (2007). Cost-effectiveness of high-dose atorvastatin compared with regular dose simvastatin. Eur Heart J.

[12] Gandhi SK, Jensen MM, Fox KM, Smolen L, Olsson AG, Paulsson T (2012). Cost-effectiveness of rosuvastatin in comparison with generic atorvastatin and simvastatin in a Swedish population at high risk of cardiovascular events. ClinicoEconomics and outcomes research : CEOR.

[13] Jernberg T, Attebring MF, Hambraeus K (2010). The Swedish web-system for enhancement and development of evidence-based care in heart disease evaluated according to recommended therapies (SWEDEHEART). Heart.

[14] SWEDEHEART (2014). Annual report 2013. http://www.ucr.uu.se/swedeheart/. Accessed 16 Oct 2016.

[15] Wilson PW, D'Agostino R, Bhatt DL (2012). An international model to predict recurrent cardiovascular disease. Am J Med.

[16] Jernberg T, Hasvold P, Henriksson M, Hjelm H, Thuresson M, Janzon M (2015). Cardiovascular risk in post-myocardial infarction patients: nationwide real world data demonstrate the importance of a long-term perspective. Eur Heart J.

[17] Ara R, Tumur I, Pandor A (2008). Ezetimibe for the treatment of hypercholesterolaemia: a systematic review and economic evaluation. Health Technol Assess.

[18] Hallberg S, Gandra SR, Fox KM, et al. Healthcare costs associated with cardiovascular events in patients with hyperlipidemia or prior cardiovascular events: estimates from Swedish population-based register data. Eur J Health Econ. 2015; doi:10.1007/s10198-015-0702-0. 10.1007/s10198-015-0702-0 PMC4869759 26077550

[19] Ara R, Pandor A, Stevens J, Rees A, Rafia R (2009). Early high-dose lipid-lowering therapy to avoid cardiac events: a systematic review and economic evaluation. Health Technol Assess.

[20] Steen-Karlsson K, and Persson U. (2012) Kostnader för Hjärt-Kärlsjukdom år 2010 IHE Rapport Lund: Institutet för Hälso- och Sjukvårdsekonomi.

[21] Danese M LM, Villa G, Lindgren P, van Hout B, and Taylor B. Differences between observed and predicted cardiovascular event rates using standard tools for risk prediction. The case of high-intensity statin users in the United Kingdom. Circulation 2015; 132: A18114 2015.

[22] Nichols M, Townsend N, Scarborough P, Rayner M (2013). Trends in age-specific coronary heart disease mortality in the European Union over three decades: 1980-2009. Eur Heart J.

[23] Nichols M, Townsend N, Scarborough P, Rayner M (2013). Cardiovascular disease in Europe: epidemiological update. Eur Heart J.

[24] Kotseva K, Wood D, De Bacquer D (2016). EUROASPIRE IV: a European Society of Cardiology survey on the lifestyle, risk factor and therapeutic management of coronary patients from 24 European countries. Eur J Prev Cardiol.

[25] SWEDEHEART (2017). Annual report 2016. http://www.ucr.uu.se/swedeheart/dokument-sh/arsrapporter. Accessed 2 March 2017.

[26] Pettersson B, Hoffmann M, Wandell P, Levin LA (2012). Utilization and costs of lipid modifying therapies following health technology assessment for the new reimbursement scheme in Sweden. Health policy.

[27] Foody JM, Joyce AT, Rudolph AE, Liu LZ, Benner JS (2008). Persistence of atorvastatin and simvastatin among patients with and without prior cardiovascular diseases: a US managed care study. Curr Med Res Opin.

[28] Ho PM, Spertus JA, Masoudi FA (2006). Impact of medication therapy discontinuation on mortality after myocardial infarction. Arch Intern Med.

[29] Ho PM, Bryson CL, Rumsfeld JS (2009). Medication adherence: its importance in cardiovascular outcomes. Circulation.

[30] Persson U, Norinder A, Hjalte K, Gralen K (2001). The value of a statistical life in transport: findings from a new contingent valuation study in Sweden. J Risk Uncertainty.

[31] Persson U, Hjelmgren J (2003). Health services need knowledge of how the public values health. Lakartidningen.

[32] Ludvigsson JF, Andersson E, Ekbom A (2011). External review and validation of the Swedish national inpatient register. BMC Public Health.

[33] Piepoli MF, Hoes AW, Agewall S (2016). 2016 European guidelines on cardiovascular disease prevention in clinical practice: the sixth joint task force of the European Society of Cardiology and Other Societies on cardiovascular disease prevention in clinical practice (constituted by representatives of 10 societies and by invited experts): developed with the special contribution of the European Association for Cardiovascular Prevention & rehabilitation (EACPR). Eur Heart J.

[34] SWEDEHEART. http://www.ucr.uu.se/swedeheart/. Accessed 3 August 2017.

